# Predictors for Survival of Patients with Squamous Cell Carcinoma of Unknown Primary in the Head and Neck Region

**DOI:** 10.3390/cancers15072167

**Published:** 2023-04-06

**Authors:** Steffen Wagner, Christine Langer, Nora Wuerdemann, Susanne Reiser, Helen Abing, Jörn Pons-Kühnemann, Elena-Sophie Prigge, Magnus von Knebel Doeberitz, Stefan Gattenlöhner, Tim Waterboer, Lea Schroeder, Christoph Arens, Jens Peter Klussmann, Claus Wittekindt

**Affiliations:** 1 Department of Otorhinolaryngology, Head and Neck Surgery, Medical Faculty, University of Giessen, 35392 Giessen, Germany; 2 Department of Otorhinolaryngology, Head and Neck Surgery, Medical Faculty, University of Cologne, 50937 Cologne, Germany; 3 Medical Statistics, Institute of Medical Informatics, University of Giessen, 35392 Giessen, Germany; 4 Department of Applied Tumor Biology, Institute of Pathology, University Hospital Heidelberg, 69120 Heidelberg, Germany; 5 Clinical Cooperation Unit Applied Tumor Biology, German Cancer Research Center (DKFZ), 69120 Heidelberg, Germany; 6 Institute of Pathology, University of Giessen, 35392 Giessen, Germany; 7 Infections and Cancer Epidemiology, Infection, Inflammation and Cancer Program, German Cancer Research Center (DKFZ), 69120 Heidelberg, Germany; 8 Department of Otorhinolaryngology, Klinikum Dortmund gGmbH, University Hospital Witten/Herdecke, 44137 Dortmund, Germany

**Keywords:** carcinoma of unknown primary (CUP), oropharyngeal squamous cell carcinomas (OPSCC), human papillomavirus (HPV), head and neck cancer, risk model, principal component analysis

## Abstract

**Simple Summary:**

Human papillomavirus (HPV) association is the most important predictor of survival in squamous cell carcinomas in the head and neck region (HNSCC). The role of HPV in cancer of unknown origin at this anatomic site (CUP_HNSCC_) is less well understood. The objective of this study was to identify prognostic classification markers in CUP_HNSCC_. Therefore, we investigated a consecutive cohort by multivariate modeling and testing for HPV DNA, mRNA, and p16^INK4a^ (p16) expression. In 31% of CUP_HNSCC_, p16 was overexpressed, and high-risk HPV DNA was detected in 18/32 (56.3%) of them, which was mostly consistent with mRNA detection. In contrast to oropharyngeal cancer, detection of p16 without additional detailed HPV testing appears to be more appropriate for the classification of CUP_HNSCC_. Three risk groups can be stratified based on performance status and p16, but additional factors may become important in future data or for cases with particular risk profiles.

**Abstract:**

Background: Human papillomavirus (HPV) status is the most important predictor of survival in oropharyngeal squamous cell carcinoma (OPSCC). In patients with cervical lymph node metastases of squamous cell carcinoma of unknown origin (CUP_HNSCC_), much less is known. Methods: We assessed a consecutive cohort of CUP_HNSCC_ diagnosed from 2000–2018 for HPV DNA, mRNA, p16^INK4a^ (p16) expression, and risk factors to identify prognostic classification markers. Results: In 32/103 (31%) CUP_HNSCC_, p16 was overexpressed, and high-risk HPV DNA was detected in 18/32 (56.3%). This was mostly consistent with mRNA detection. In recursive partitioning analysis, CUP_HNSCC_ patients were classified into three risk groups according to performance status (ECOG) and p16. Principal component analysis suggests a negative correlation of p16, HPV DNA, and gender in relation to ECOG, as well as a correlation between N stage, extranodal extension, and tobacco/alcohol consumption. Conclusions: Despite obvious differences, CUP_HNSCC_ shares similarities in risk profile with OPSCC. However, the detection of p16 alone appears to be more suitable for the classification of CUP_HNSCC_ than for OPSCC and, in combination with ECOG, allows stratification into three risk groups. In the future, additional factors besides p16 and ECOG may become important in larger studies or cases with special risk profiles.

## 1. Introduction

Metastases from which the primary site is unknown are called carcinoma of unknown primary (CUP). In head and neck cancers, the most common histological type is squamous cell carcinoma (HNSCC), which is also the case in 53–90% of CUP in this anatomical region [1,2,3]. Histologic features and specific patterns of metastasis [4], particularly concerning the involved lymphatic drainage pathways [5], may reveal the likely anatomic origin of the primary tumor in the head and neck region [3]. Therefore, corresponding tumors infiltrating cervical lymph nodes in the head and neck may be considered CUP_HNSCC_, provided that other etiologies are excluded.

Infection with high-risk (HR) human papillomavirus (HPV) is causative for a subgroup of HNSCC, particularly oropharyngeal squamous cell carcinoma (OPSCC). An increasing incidence of HPV-related HNSCC is reported in several countries worldwide [6,7,8]. In CUP_HNSCC_, a substantial proportion of cases also appear to be associated with HPV [9,10,11]. During HPV-driven carcinogenesis, key features of apoptosis and cell cycle control mechanisms are deregulated by viral oncoproteins. A consequence of this and the hallmark of HPV-driven carcinogenesis is overexpression of the cellular tumor suppressor protein cyclin-dependent kinase inhibitor 2A, also known as p16^INK4a^ (p16). Overexpression of p16 has been introduced in the AJCC-8/UICC-8 staging system as a surrogate marker for classifying HPV-associated OPSCC and CUP_HNSCC_ [12].

The prognosis is significantly better in HPV-related than in HPV-negative OPSCC, and risk models have shown that HPV is the most important prognosticator in these patients [13,14,15,16]. It is also known that the prognosis for HPV-related compared with HPV-negative CUP_HNSCC_ is considerably better [11,17,18,19]. Furthermore, CUP_HNSCC_ and OPSCC are thought to be related diseases [10] because both cancers have similar risk factors and develop from epithelial squamous cells. However, comparative data are sparse. In OPSCC, the replacement of cisplatin with cetuximab failed in cisplatin-based chemoradiotherapy [20,21]. Nevertheless, ongoing clinical phase I–III trials are investigating a de-escalating treatment for this patient group [22]. In 5–20% of p16-positive OPSCC, no HPV–DNA or mRNA is detectable [23,24,25,26,27], and we have shown that p16 as a single marker may not be sufficient to identify OPSCC patients suitable for treatment de-escalation [27]. In a systematic review from 2019 [11], the overall rate of an HPV association was estimated to be 40~60% in CUP patients. However, in 8/17 of the included studies, only a single marker (in most cases p16) was used to test for HPV. The rate decreased to 17 and 39%, respectively, when HPV mRNA or DNA was also tested in addition to p16. Moreover, remarkable heterogeneity regarding the HPV-positive rate by any method was found between North America (58%) and Europe (34%), as well as within studies from the same continent [11].

In this context, we aimed to determine the HPV prevalence in CUP_HNSCC_ by analyzing p16 expression and detecting HPV DNA and mRNA. In addition, we sought to identify prognostic classification markers in CUP_HNSCC_ multivariate using recursive partitioning (RPA) and principal component analysis (PCA) [28,29] to identify a potential association of factors and the most important predictors for survival in CUP_HNSCC_.

## 2. Materials and Methods

Patients: We retrieved clinical data of all patients diagnosed with CUP_HNSCC_ (according to ICD10) and treated at our hospital (Department of Otorhinolaryngology, Head and Neck Surgery of the University Hospital Giessen) between 2000 and 2018. The study was performed retrospectively in accordance with the local ethics committee (AZ 151/11; 296/11). Exclusion criteria were an absence of written informed consent, histologically confirmed primary tumor outside the cervical lymph nodes, or unavailable or insufficient amounts of formalin-fixed, paraffin-embedded samples from the pre-therapeutic tumor biopsy or tissue from tumor resection. The absence of primary tumors in the head and neck region was confirmed by panendoscopy with bilateral diagnostic tonsillectomy and biopsies from the nasopharynx and base of the tongue. In addition, all patients since 2011 received pretherapeutic ^18^F-fluorodeoxyglucose positron emission tomography/computed tomography as part of the diagnostic workup. Follow-up examinations included systematic inspection of the entire oral cavity, oropharynx and neck, ultrasonography of the cervical lymph nodes, and computed tomography or magnetic resonance imaging scans at regular intervals. All examinations were performed according to the guidelines of the German Guideline Program in Oncology (GGPO) valid at the time of the examinations.

Therapy and risk factors: CUP_HNSCC_ was treated by upfront surgery (neck dissection) and/or by radio- (RT) or chemoradiotherapy (RCT) (Table 1). Treatment decisions were in accordance with local guidelines and were made after discussing each case in an interdisciplinary tumor board and patient decision. Regarding chemotherapy as part of curative treatment, patients treated with RCT only (*n* = 4) received cisplatin or mitomycin C (MMC) and 5-fluorouracil (5-FU) in two cases each. Patients treated by surgery and adjuvant RCT (*n* = 37) received cisplatin or carboplatin (*n* = 23, *n* = 2), MMC + 5FU (*n* = 4) or 5-FU + Cisplatin (*n* = 5). In three cases, the chemotherapeutic agent was not specified.

When patients presented, tumors were classified by pathological or clinical stages (if surgical resection was not performed) according to the International Union Against Cancer (UICC) TNM classification valid at the time of diagnosis [30,31]. The N stages used in this work correspond to the following classification: N1, metastasis in a single ipsilateral lymph node, ≤3 cm in greatest dimension; N2a, metastasis in a single ipsilateral node, >3 cm but ≤6 cm in greatest dimension; N2b, metastasis in multiple ipsilateral nodes, none > 6 cm in greatest dimension; N2c, metastasis in bilateral or contralateral nodes, none > 6 cm in greatest dimension; N3, metastasis in a lymph node > 6 cm in greatest dimension. Classification of extranodal extension (ENE) was based on radiological or pathological findings if resected lymph node tissue was available. Histological grading was performed according to the WHO criteria for squamous cell carcinomas of the oral mucosa [32]. Cases other than squamous cell carcinomas were excluded. Records of the Giessen cancer registry database (GTDS) and patient charts were reviewed for tumor characteristics, risk factors, and therapy. By dichotomization, the following categories were formed: smokers and non-smokers if they had >10 or ≤10 pack-years during, respectively, in the past 16 years; alcohol consumption (drinkers) and no alcohol consumption (non-drinkers) if they had >2 and ≤2 standard drinks, respectively, on average each day; low to moderate (≤2) or high (>2) histopathological grading; good to moderate or poor performance status according to the Eastern Cooperative Oncology Group (ECOG) 0–1 vs. 2–4.

HPV status: HPV status of CUP_HNSCC_ was determined by immunohistochemical detection of p16 expression using the CINtec Histology Kit (Roche mtm Laboratories) and by analyzing DNA extracted from archived formalin-fixed, paraffin-embedded tumor tissue for mucosal high-risk (HR)-HPV DNA, as we described previously [7,33]. The Luminex bead-based HPV detection and genotyping kit (Multiplex HPV Genotyping Kit for Research in Epidemiology, Multimetrix, Heidelberg, Germany) allows for the detection of 15 high-risk types (16, 18, 31, 33, 35, 39, 45, 51, 52, 56, 58, 59, 68, 73, and 82), three putative high-risk types (26, 53, and 66) and six low-risk types (6, 11, 42, 43, 44, and 70). Strong, diffuse expression in >70% of tumor cells was considered positive for p16. Samples with insufficient amounts and/or insufficient integrity of the extracted DNA, as determined by PCR amplification with the included β-globin primers, were excluded from the analysis. HR-HPV mRNA analysis was performed by HPV type-specific reverse transcription polymerase chain reaction (RT-PCR) and hybridization assays (developed for HPV types: 16, 18, 26, 31, 33, 35, 39, 45, 51, 52, 53, 56, 58, 59, 66, 67, 68b, 70, 73, and 82) as described previously [10,34]. All available samples that tested positive for p16 and/or HR-HPV DNA were analyzed, as well as control samples (*n* = 20) that tested negative for both. Briefly, amplification of HPV E6*I (approximately 65 base pairs) was used to determine HPV transcriptional activity, and ubiquitin C (85 base pairs) was used as a cellular marker to control mRNA quality and confirm assay validity.

Statistics: Recursive partitioning analysis (RPA) was performed by using the function ctree (conditional inference trees) of the R (Version 4.0.4) package partykit, Version 1.2–12 [35]. The stop criterion was set to multiplicity-adjusted *p*-values (test type = ‘Bonferroni’). Principal component analysis (PCA) [28,29] was used as an eigenvector-based multivariate method to examine the internal structure of our data. The Varimax orthogonal rotation method was performed for PCA. Overall survival (OS, calculated from the date of diagnosis to the date of death from any cause or date of last seen alive) was used to plot survival curves by the Kaplan–Meier method. SPSS Statistical Software (IBM SPSS 27.0) was used for statistical analysis. Significance was considered *p* ≤ 0.05 for all tests unless otherwise indicated. With the exception of RPA, *p*-value adjustment for multiple testing was not performed due to the exploratory nature of the study.

## 3. Results

### 3.1. Descriptive Analysis of CUP_HNSCC_ according to p16 Status

Tumor characteristics, lifestyle- and patient-related risk factors for CUP_HNSCC_ stratified by p16 status are shown in Table 1. Patients with p16-positive CUP_HNSCC_ were less likely to have increased tobacco and alcohol consumption, advanced N stage (>2b), and extranodal extension (ENE). In contrast, our data show an increased male-to-female ratio and worse performance status (ECOG) in patients with p16-negative CUP_HNSCC_, although without reaching statistical significance (Table 1). A curative treatment option could not be offered (or the patients refused treatment) to 12/71 (17%) patients with p16-negative and 1/32 (3%) with p16-positive CUP_HNSCC_ (*p* = 0.057). When treated with curative intent, most cases underwent upfront surgery with adjuvant radio- (35/88, 40%) or chemoradiotherapy (37/88, 42%), with no significant differences according to p16 status (Table 1).

### 3.2. HPV Status in CUP_HNSCC_ and Survival of Patients

**Figure 1:**
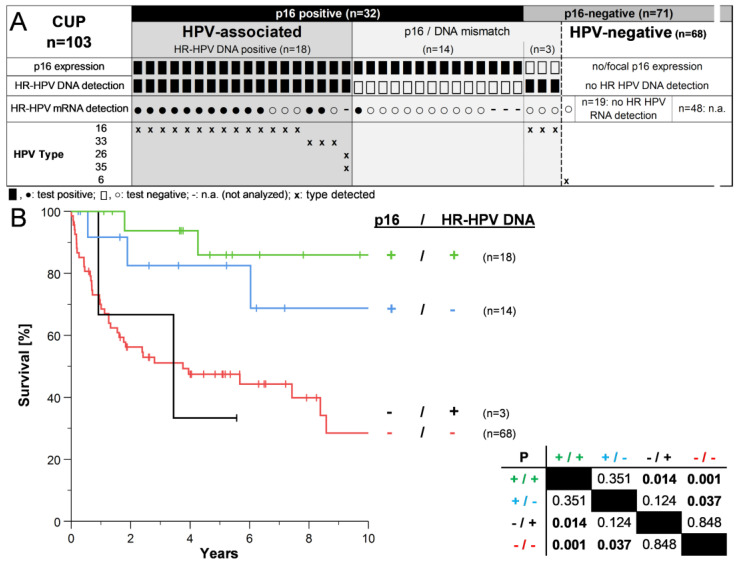
HPV status and overall survival (OS) of patients with cancers of unknown primary in the head and neck region (CUP_HNSCC_). (**A**): HPV status of CUP_HNSCC_ determined by detecting p16^INK4a^ (p16) expression and HR-HPV DNA and mRNA. (**B**): OS of patients with CUP_HNSCC_ stratified by HPV status; censored cases indicated with vertical markers. *p*: *p*-value (log-rank test).

Overexpression of p16 was found in 32/103 (31%) samples (Figure 1A). The incidence of CUP_HNSCC_ overall and stratified by p16 status did not change significantly over time (5.4 cases overall annually, linear regression). HR-HPV DNA was detected in 21/103 (20.4%) of all samples but not in 14/32 (43.8%) of p16-positive samples. HR-HPV mRNA assays confirmed the DNA-based results in most cases. The mRNA assay was valid in all samples analyzed but did not detect HPV mRNA in seven samples positive for HR-HPV DNA (Figure 1A, open circles). Only in one sample with p16 overexpression and undetectable HPV DNA was HR-HPV mRNA detected. The HPV type matched in all cases with valid DNA and mRNA tests. HPV16 was the most abundant HPV type found in 17/21 (81.0%) samples with detectable HR-HPV DNA. Only one case (without p16 overexpression) was positive for a low-risk HPV type (Figure 1A). Considering p16 overexpression as the reference, testing for HR-HPV DNA in CUP_HNSCC_ reached a sensitivity of 56.3%, a specificity of 95.8%, and an accuracy of 83.5%. The false negative and false positive rates were 43.8% and 4.2%, respectively. Concerning the samples investigated, HR-HPV mRNA testing reached a sensitivity of 50%, a specificity of 100%, and an accuracy of 72.5%. The false negative and false positive rates were 50% and 0%, respectively.

Overexpression of p16 was associated with remarkably improved OS in patients with CUP_HNSCC_ (Figure 1B). Five-year (5Y) OS rates were about 80% compared with less than 50% in patients with p16-negative CUP_HNSCC_. This was true regardless of HR-HPV DNA. Patients with p16-positive CUP_HNSCC_ and detectable as well as undetectable HR-HPV DNA had significantly better OS compared with CUP_HNSCC_ negative for both markers (*p* = 0.001 and *p* = 0.037, Figure 1B).

### 3.3. Development of a Risk Model for CUP_HNSCC_ by Recursive Partitioning Analysis (RPA)

**Figure 2:**
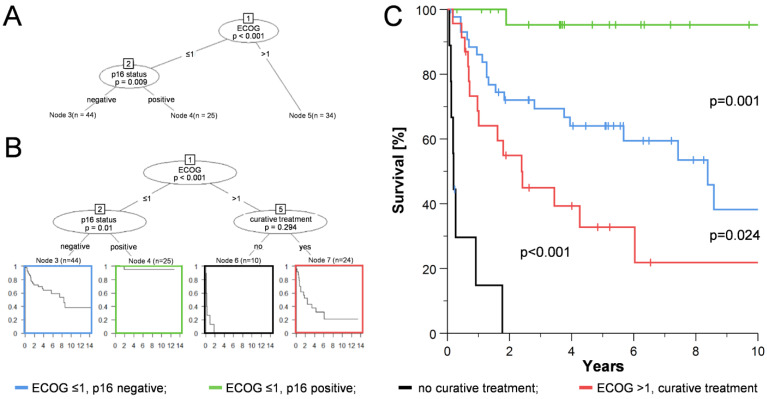
Development of a risk model for CUP_HNSCC_. (**A**): Multivariate analysis of patient characteristics and risk factors by recursive partitioning (RPA). Included factors: p16 status (negative/positive), HR-HPV DNA detection (negative/positive), gender (female/male), age (years), N stage (1–3), extranodal extension (no/yes), tobacco (no/yes) and alcohol (no/yes) consumption, performance status (ECOG 0–4), and histological grading (low: ≤2/high: >2). The splitting threshold was set to 0.05, and the *p*-values calculation was set to “Bonferroni”. Only splitting at nodes 1 (ECOG) and 2 (p16) resulted in significant differences in the OS of subgroups. (**B**): As figure A only with the addition of curative treatment possibility (no/yes) in the RPA and splitting threshold set to 0.5 to allow for node formation without significant difference in OS of the resulting groups. (**C**): OS of patients with CUP_HNSCC_ stratified by the RPA model generated in (**B**). *p*: *p*-value (log-rank test).

We performed recursive partitioning to multivariately investigate the impact of risk factors on OS and to build a risk model for patients with CUP_HNSCC_ (Figure 2). All relevant patient characteristics and risk factors from Table 1 were included, except for treatment, as this highly depends on tumor and patient characteristics/decisions. The conditional interference tree developed a robust structure suggesting that performance (ECOG) and p16 status were the most important predictors of OS in patients with CUP_HNSCC_ (Figure 2A). Here, a threshold between 1 and 2 of ECOG status showed the best possible separation. HR-HPV DNA detection, gender, age, extranodal extension, histological grading, tobacco, and alcohol did not reach a rank to be displayed in the hierarchical clustering.

Classification of CUP_HNSCC_ into three risk groups according to ECOG and p16 status (Figure 2A) is theoretical, as it does not consider whether or not treatment (with curative intension) is possible. Therefore, as a next step, we included the treatment option (curative or not) in the RPA, which is then reported as another option for group separation in patients with ECOG > 1 (Figure 2B), although not significant in this modeling. Considering the need to distinguish between patients with and without a curative treatment option, classification by ECOG and p16 status still leads to a clear separation of the three proposed risk groups (Figure 2C).

### 3.4. Correlation of Factors Affecting the Formation of the Risk Model in Patients with CUP_HNSCC_

**Figure 3:**
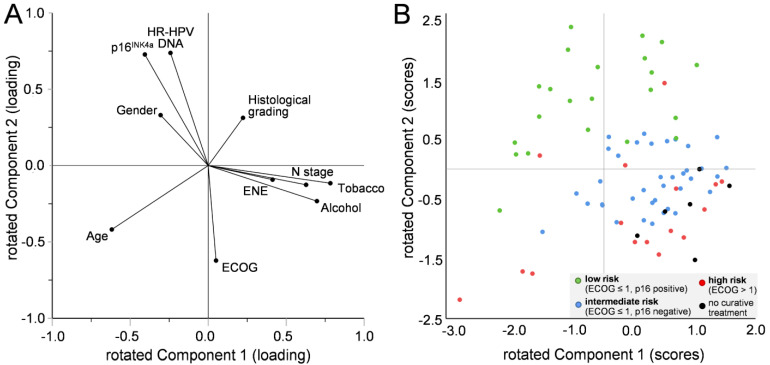
Principal component analysis (PCA) of tumor characteristics and lifestyle/patient-related risk factors in CUP_HNSCC_ and distribution of cases according to the resulting two main components (components 1 and 2). (**A**): Loading plot of components 1 and 2 using the indicated variables (further details see text and Table 1). (**B**): Distribution of all cases with CUP_HNSCC_ without missing data (*n* = 81) according to the resulting main component scores in comparison to risk groups (green, red, and blue) predicted by recursive partitioning (RPA) and non-curative treatment (black).

We used principal component analysis (PCA) to capture the most important aspects of the data by reducing complexity and to assess possible relationships between factors independent of survival. All factors previously used for the RPA were included, and we used the first two components (1 and 2) for a two-dimensional visualization (Figure 2A). The loading of all factors is plotted in Figure 3A. In brief, a vector is formed for each factor, whose direction and length from the origin indicate the respective contribution to the two components. A possible relationship between the factors is characterized by the angle between the respective vectors (a small angle represents a probable positive correlation, ~180° a probable negative correlation, and almost 90° an unlikely correlation). Figure 3A suggests a correlation between p16 expression, positivity for HR-HPV DNA, and gender, which negatively correlates with ECOG. N stage clusters with ENE, tobacco, and alcohol consumption. The vectors of age and histological grading show an opposite orientation, suggesting a negative correlation with each other and no likely correlation with p16 expression, HR-HPV DNA, and gender.

In PCA, a value for components 1 and 2 was calculated for each individual case, describing its position in a two-dimensional space (Figure 3B). In simplified terms, the position of each data point in Figure 3B is based on the direction and length of the vectors in Figure 3A and their contribution to each patient’s risk profile. Cases top left in Figure 3B, for example, have a very low-risk factor profile. The more a case is oriented to the bottom right of Figure 3B, the more likely are negative influences of additional risk. Example cases are shown in detail in Supplementary Figure S1.

## 4. Discussion

We found that 31% of CUP_HNSCC_ in our consecutive cohort overexpressed p16, but HPV-related carcinogenesis was confirmed by DNA detection in only 17.5% of all cases. This discrepancy is remarkable but also known from the literature. For example, in a systematic review, the estimated rate of HPV association in CUP patients decreased from 40~60% to 17 and 39%, respectively, when HPV mRNA or -DNA was tested in addition to p16 [11]. Compared with the literature, the rate of p16 overexpression and “truly” HPV-related cases in our cohort is rather low. However, a remarkable heterogeneity in terms of geographical regions is known [11], and our data are comparable to other studies from Central Europe reporting similar rates [17,36,37,38,39].

It is known that during surgical treatment of HPV-positive lesions, equipment, and protective clothing can become contaminated with HPV [40]. Compared to OPSCC [27], we found fewer samples with detectable HR-HPV DNA but lacking p16 expression in CUP_HNSCC_—possibly due to a lower probability of contamination during resection of “sterile” lymph nodes compared with samples from the oral cavity, which could still contain productive HPV infections. A lack of detection of HR-HPV DNA in p16-positive samples, on the other hand, could be due to the reduced integrity of DNA in archived samples. However, we excluded samples in which the β-globin gene could not be amplified, so the effect of aging should be small. This is confirmed by our results of the HR-HPV mRNA assay. Although mRNA is more unstable than DNA, the assay was valid in detecting the control mRNA in all samples analyzed. Only in one case with p16 overexpression was HR HPV-mRNA detectable, but no DNA. This does not particularly support the assumption that these cases are indeed HPV-associated. Despite the relatively small size of our cohort, the overall performance of the two assays (HR-HPV DNA and mRNA) was reasonably comparable, and their results largely agreed. This argues against fundamental technical/methodological problems in detecting HPV in archival samples. On the other hand, we might assume that p16 expression in the absence of HR-HPV DNA may be due to the nature of carcinogenesis in CUP_HNSCC_. HPV-independent overexpression of p16 is known from other tumors [41,42,43] for still unknown reasons. Another possibility could be that a small (undetectable) primary appears to spread to regional lymph nodes much earlier than in OPSCC. At this early stage, loss of HR-HPV DNA (or sections of the viral genome relevant to HPV diagnosis) and the development of (putative) HPV-independent carcinogenesis could be more common, although this is only speculation. It is more likely that the number of tumor cells and, thus, the amount of HR-HPV DNA in some CUP_HNSCC_ samples is quite low (but sufficient for detecting the control PCR product) and, therefore, may not be sufficient for the detection of HPV. This would imply that at least some of the p16-positive/HR-HPV DNA-negative samples are positive for both, i.e., HR-HPV DNA false-negative. This is also supported by our survival data (Figure 1B) but not by our mRNA assay data (Figure 1A). However, this assumption is supported by a study of a prospective clinical cohort. Tissue was available from *n* = 49 CUP_HNSCC,_ and only two samples negative for HR-HPV DNA or mRNA were p16 positive [10]. Although the detection of p16 alone seems more suitable for the classification of CUP_HNSCC_ than for OPSCC, we currently do not consider it reasonable to omit the detection of HPV DNA or mRNA itself in clinical diagnostics. However, future studies will clarify this issue, as assessment of HPV status in patients with CUP_HNSCC_ is now indicated using the AJCC-8/UICC-8 staging system [12]. Thus, the necessary data will become available.

In most cases of cervical lymph node metastases where another primary origin can be ruled out, and especially in HPV-associated CUP_HNSCC_, it is likely that the tumors arose from epithelial cells in the head and neck. Therefore, we expected that similar risk factors would be important for survival in CUP_HNSCC_ as we have previously shown for OPSCC [16,27]. In the risk model for CUP_HNSCC_, ECOG, and p16 (in this case as the only marker for HPV status) are the most important factors and sufficient to stratify patients into three risk groups with significant differences in OS. This is consistent with our previous results for OPSCC, although HPV DNA/RNA status is more important there. In subjects with good to moderate performance and p16 negative CUP_HNSCC_, we could not verify N status as another important factor, as was the case in OPSCC [16]. The relatively small size of our cohort limits the classification of additional CUP_HNSCC_ subgroups. Nevertheless, PCA shows that ENE, smoking, and alcohol consumption correlate with N status, which is likely the next important factor in RPA. We acknowledge that the RPA allowed us to identify only the most important factors here, but this does not preclude the identification of other relevant factors when larger cohorts become available in future analyses.

We found some, although not significant, discrepancies in treatment intensity between patients with p16-positive and -negative CUP_HNSCC_ (Table 1). This is an important aspect that may bias survival data. Although this factor is essential for patient prognosis, it appears only secondarily in the RPA analysis (Figure 2) and did not change the overall model. This could be because several other factors are related to the treatment choice, and therefore, it plays a minor role in the RPA analysis. This is consistent with our findings from PCA (which does not depend on survival data), showing that patients who could not be offered a curative treatment option cluster in the lower right sector (Figure 3B). Patient age at diagnosis did not appear in the risk model for CUP_HNSCC_. This is somewhat surprising since with increasing age, the number and severity of comorbidities usually increase, and overall performance status decreases. This is expected to impact both treatment choice and outcome [44]. However, this is consistent with our data from OPSCC, suggesting that overall performance status is more important than the physical age of the patient. Also, this is consistent with the suggestion that not only chronological but also biological age and functional status (and probably other factors) should be considered when selecting the best cancer treatment [45,46,47].

The main limitation of this study is the overall small sample size of our CUP_HNSCC_ cohort. Nevertheless, this is comparable (and even higher) with similar published cohorts [11,48], and the incidence of CUP_HNSCC_ is rare (less than 5% of all head and neck tumors) [10,49,50]. In this regard, it is of particular importance that our cohort represents a temporally and spatially consistent sample that was treated equivalently to the corresponding OPSCC cohort, and we performed a comprehensive analysis of HPV status (p16, DNA, and mRNA). Interestingly, the PCA in OPSCC [27] and CUP_HNSCC_ show some differences: we found a negative correlation between ECOG and p16 status for CUP_HNSCC_, whereas, in OPSCC, a correlation seems overall unlikely. On the other hand, ECOG correlated together with T- (and N stage) in OPSCC, which cannot be investigated for CUP_HNSCC_. In CUP_HNSCC_, we found a likely correlation between the N stage, along with tobacco and alcohol use, which was unlikely in OPSCC [27].

## 5. Conclusions

The role of HPV and risk factors in CUP_HNSCC_ is not completely understood. In this consecutive cohort analyzed for HPV DNA, mRNA, and p16 expression, and by multivariate modeling, we found that CUP_HNSCC_ patients could be classified by performance status (ECOG) and p16. Despite obvious differences, CUP_HNSCC_ shares similarities in risk profile with OPSCC. In contrast to OPSCC, the detection of p16 alone (without additional HPV testing) appears more appropriate for the classification of CUP_HNSCC_. Three risk groups of patients with CUP_HNSCC_ and significant differences in OS can be stratified based on ECOG and p16, though additional factors may become important in future studies or cases with particular risk profiles.

## Figures and Tables

**Table 1:** Descriptive analysis of CUP_HNSCC_ according to p16 status.

		Total		p16-Positive		p16-Negative		*p*
		*n*	*%*	*n*	*%*	*n*	*%*	
Total		103	*100*	32	*31*	71	*69*	N/A
Gender	Male	78	*76*	21	*66*	57	*80*	0.108
	Female	25	*24*	11	*34*	14	*20*	
Age (years)	Median	62.9		62.0		63.0		0.847 #
	Minimum	32.2		47.0		32.2		N/A
	Maximum	95.2		84.9		95.2		N/A
Sampling period	2000 to 2009	50	*49*	15	*47*	35	*49*	0.820
	2010 to 2018	53	*51*	17	*53*	36	*51*	
N stage	1	11	*11*	3	*9*	8	*11*	0.168
	2a	15	*15*	7	*22*	8	*11*	
	2b	48	*47*	18	*56*	30	*43*	
	2c	5	*5*	1	*3*	4	*6*	
	3	23	*22*	3	*9*	20	*29*	
	unknown	1	*1*	-		1		
N stage (dichotomized)	1–2b	74	*73*	28	*88*	46	*66*	**0.022**
	>2b	28	*27*	4	*13*	24	*34*	
Extranodal extension (ENE)	No	35	*34*	15	*58*	20	*33*	**0.035**
	Yes	51	*50*	11	*42*	40	*67*	
	unknown	17	*17*	6		11		
Tobacco	Non-smokers	25	*24*	13	*43*	12	*18*	**0.007**
	Smokers	73	*71*	17	*57*	56	*82*	
	unknown	5	*5*	2		3		
Alcohol	Non-drinkers	55	*53*	24	*77*	31	*45*	**0.003**
	Drinkers	45	*44*	7	*23*	38	*55*	
	unknown	3	*3*	1		2		
ECOG	0	16	*16*	7	*22*	9	*13*	0.469
	1	53	*51*	18	*56*	35	*49*	
	2	26	*25*	6	*19*	20	*28*	
	3	7	*7*	1	*3*	6	*8*	
	4	1	*1*	0	*0*	1	*1*	
ECOG (dichotomized)	0–1	69	*67*	25	*78*	44	*62*	0.107
	>1	34	*33*	7	*22*	27	*38*	
Histological grading								
low/intermediate	1–2	38	*37*	8	*25*	30	*43*	0.083
high grade	>2	64	*62*	24	*75*	40	*57*	
	unknown	1	*1*	-		1		
Treatment intension	Palliative	13	*13*	1	*3*	12	*17*	0.057 *
	Curative	88	*85*	31	*97*	57	*83*	
	unknown	2	*2*	-		2		
Curative treatment (% based on *n* = 88)	Surgery only	8	*9*	2	*7*	6	*11*	0.730
	Surgery + RT	35	*40*	13	*43*	22	*39*	
	Surgery + RCT	37	*42*	12	*40*	25	*44*	
	RT only	3	*3*	2	*7*	1	*2*	
	RCT only	4	*5*	1	*3*	3	*5*	
	unknown	1	*1*	1		-		
Median follow-up (years)		2.8		5.3		1.9		N/A

*p*: Pearson’s chi-squared test; *: Fisher´s exact test, 2-sided; #: Mann-Whitney-U-test; RT: radiotherapy; RCT: chemoradiation; p16: p16^INK4a^; percentages in italics; significant *p*-values in bold; N/A: not applicable.

## Data Availability

With regard to potentially personalized data, ethical, and legal rules, data can be made available upon reasonable request from the corresponding author for academic research within the constraints of the consent given by the patients.

## Supplementary Materials

The following supporting information can be downloaded at: https://www.mdpi.com/article/10.3390/cancers15072167/s1, Figure S1: Examples of patients with CUP_HNSCC_ stratified by principal component analysis (PCA).
