# Supplementary material for: Predictors for Survival of Patients with Squamous Cell Carcinoma of Unknown Primary in the Head and Neck Region
Source: Cancers (Basel). 2023 Apr 6;15(7):2167. doi: 10.3390/cancers15072167 (PMC10093301; doi:10.3390/cancers15072167)
Supplement: Supplementary file 1 [file cancers-15-02167-s001.zip › cancers-2221512-supplementary.pdf]

Supplementary Figure S1: Examples of patients with CUP_{HNSCC} stratified by principal component analysis (PCA).

A

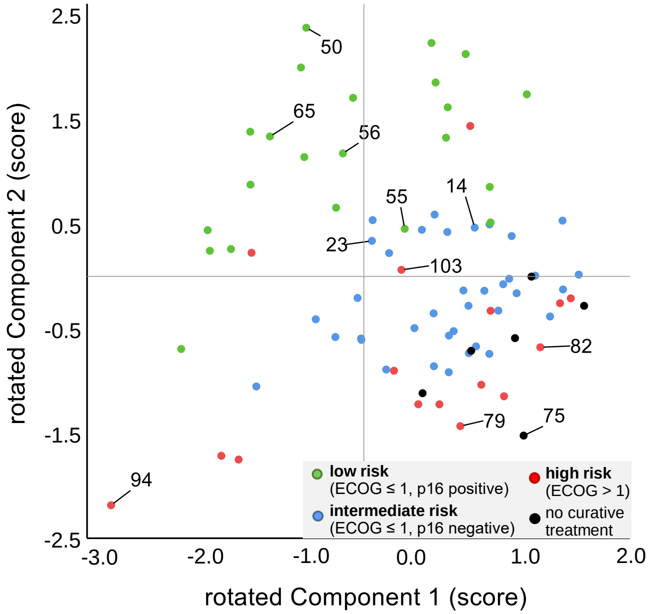

B

RPA Risk group	No.	ECOG	p16 / HR DNA	N stage	Gender	ENE	Smoking & Alcohol	Age	Hist. grading	Status	OS
**low** ECOG ≤1 p16+	50	0	+ / +	N2a	f	no	no / no	51.3	high	alive	5.2
	65	1	+ / +	N1	m	no	no / no	59.4	high	alive	12.7
	56	1	+ / +	N2a	m	no	yes / no	69.8	high	alive	7.8
	55	1	+ / -	N2c	m	no	yes / no	65.0	high	alive	6.2
**intermediate** ECOG ≤1, p16-	23	0	- / -	N2b	f	no	yes / no	68.9	high	alive	5.7
	14	1	- / -	N2b	f	yes	yes / no	35.9	low	dead	2.8
**high** ECOG >1	103	2	+ / -	N1	m	no	yes / yes	59.8	high	alive	5.2
	94	3	- / -	N1	f	no	no / no	90.1	low	alive	1.9
	82	2	- / -	N3	m	yes	yes / yes	62.6	high	dead	0.7
	79	3	- / -	N3	m	no	yes / yes	72.7	high	dead	0.7
no curative treatment	75	4	- / -	N3	m	yes	yes / yes	61.8	high	dead	0,2

A: Distribution of all cases with CUP_{HNSCC} without missing data (n =81) according to the resulting two main components (components 1 and 2) of the principal component analysis shown in Figure 3, color-coded according to the predicted risk groups (Figure 2C). Selected cases labeled. **B:** Risk factor profile heatmap of selected cases (No.: labeled in A) in comparison to risk groups predicted by recursive partitioning (RPA) and overall survival (OS).
